# **The complex intervention day hospice — a quality-assured study on the implementation, realization, and benefits with model character for Germany (IMPULS) using the example of “Day hospice Adiuvantes”**

**DOI:** 10.1186/s12904-024-01346-1

**Published:** 2024-01-16

**Authors:** Ulrich Kaiser, Ursula Vehling-Kaiser, Ana Hoffmann, Moritz Fiedler, Alexandra Hofbauer, Michael Rechenmacher, Anne Benning, Michael Koller, Florian Kaiser

**Affiliations:** 1 Clinic and Polyclinic for Internal Medicine III, University Hospital Regensburg, Regensburg, Germany; 2 Oncology–Palliative Care Network Landshut, Landshut, Germany; 3 VK&K Studien GbR, Landshut, Germany; 4 Day hospice Adiuvantes, Vilsbiburg, Germany; 5 Center for Palliative Medicine, University Hospital Regensburg, Regensburg, Germany; 6 Center for Clinical Studies, University Hospital Regensburg, Regensburg, Germany

**Keywords:** Day hospice, Complex intervention, Structural requirements, Effect, Everyday conditions

## Abstract

**Background:**

Currently, a conclusive experience on the uniform implementation and benefits of day hospice structures and interventions is lacking in Germany. The following questions should be clarified: (1) Which structural conditions and interventional measures should be established in day hospices from the point of view of patients, relatives, and specialist staff?; (2) Are the planned structures or interventions feasible and implementable under real conditions and accepted by patients, relatives, and staff?; (3) How can a final implementation and intervention catalog for day hospices be designed?; (4) Is this final catalog of services feasible, reasonable, economical, and effective under everyday conditions in day hospices?

**Methods:**

We planned to perform a multistage investigation, guided by the Medical Research Council Framework for the development and evaluation of complex interventions. In Stage 1, an initial theoretical construct on structures and interventions will be established through an extensive literature and guideline review on day hospices and through qualitative interviews. In a nominal group process, we will create a catalog of offers. In Stage 2, feasibility testing is conducted in a single-day hospice under real-life conditions using quantitative quality indicators and qualitative interviews. Structures and interventions can be adapted here if necessary. In a second nominal group process, a final structure and offer catalog is created, which is then implemented in Stage 3 in the day hospice under investigation and evaluated under real daily conditions through a process and effectiveness test. For this purpose, qualitative and quantitative quality indicators will be used and a comparative cohort of patients who are not cared for in the day hospice – but in the same network structure (oncology–palliative care network Lower Bavaria) – is examined.

**Discussion:**

Finally, the initial statements on the reasonable and realizable structures or interventions in day hospices and their benefits in daily real-life conditions as well as possible optimization processes shall be made.

**Trial registration:**

The study was retrospectively registered in the German Clinical Trials Register (DRKS-ID DRKS00031613, registration date April 04, 2023) and the display portal of the Center for Clinical Trials of the University Hospital Regensburg (Z-2022-1734-6, registration date July 01, 2023).

## Background

Day hospices, as another form of outpatient specialist palliative care, is aimed at, among other things, promoting “independence and (…) quality of life of people with a life-limiting illness, primarily through social interaction (group offerings, artistic offerings, café, and so on), rehabilitation, physiotherapy, occupational therapy, artistic therapies (art therapy, music therapy, etc.), and/or medical care for symptom control” [1]. Another focus of day hospices is respite care. The hourly or daily care of palliative patients by a day hospice allows family caregivers to meet their own needs and stabilizes the family environment, thereby ensuring care of family members at home [2–4].

Previous studies have shown a heterogeneous picture of the study methodology and day hospice structure and effects [1]. Several reviews have also shown evidence of high patient satisfaction in day hospices [2, 5], primarily due to social support experienced, restful environment offered by a day hospice, and opportunities for social interaction. Although various quantitative surveys did not demonstrate a global improvement in quality of life, attendance at a day hospice led to lower symptom burden and higher level of emotional well-being in sub-aspects [6, 7]. In sum, the limited (quantitative) evidence in the literature does not yet provide a conclusive assessment of the effect of day hospices on symptom control and quality of life. However, early evidence on the potential benefit of day hospices to palliative care patients exists. Due to the limited transferability of international studies to the German health care system, the heterogeneous implementation of day hospice structures in individual studies and the partly different study quality, a direct transfer of structures, offers, and effects of day hospices, in the German health care system is not possible [1, 8, 9]. However, the positive effects resulting from social interactions [2, 5] have already been demonstrated in Germany as well; specifically the participation in social life is often difficult among palliative patients. The introduction of joint leisure activities (“palliative excursions”) in terms of “Adventure Therapy” led to a temporary strengthening of social interaction and quality of life [10, 11]. There are also first indications for day hospices in Germany, with both patients and their relatives expecting benefits from the introduction of day hospices [12]. Additionally, theoretical recommendations for the establishment and expansion of day hospices in Germany have now been published [8, 13].

Thus far, only a few day hospices exist in German-speaking countries, unlike in Anglo-Saxon countries, where they are already widely established in the medical field [2, 4, 14, 15]. Systematic planning for the establishment of day hospices does not yet exist [8]. The experience and knowledge of the effects and structures of day hospices in Germany under everyday conditions have not been extensively studied [9]. In April 2022, the “Day Hospice Adiuvantes,” which is one of the few independently operated day hospices in Germany, was opened in Vilsbiburg (Lower Bavaria, Germany). The first valid data from real (palliative-) medical and (palliative-) nursing care situations will be obtained in our planned study. Particularly, the structural and interventional framework for the establishment of a day hospice will be systematically and structurally elaborated and tested under daily conditions. For this purpose, the acceptance, adherence, feasibility, and effectiveness of the developed offers will be tested based on reliable data and quality indicators.

## Methods and study design

### Aims of the study

To clarify the fundamental questions of effectiveness, structure, and implementation in real-life practice, a scientific accompanying program is planned during the first 3 years of operation of the “Day hospice Adiuvantes.” Particularly, the following questions will be clarified:

1. Which structural conditions and interventional measures should be established in the day hospice from the point of view of patients, relatives, and specialist personnel?
2. Are the planned structures or interventions from question 1 feasible under real-life conditions, implementable, and accepted by the affected patients, relatives, and providers?
3. How can, based on the results of questions 1 and 2, a final implementation and intervention catalog for the day hospice be designed?
4. Is the final catalog of services developed under question 3 feasible, reasonable, economical, and effective under everyday conditions in the day hospice?

### Study design

**Fig. 1:**
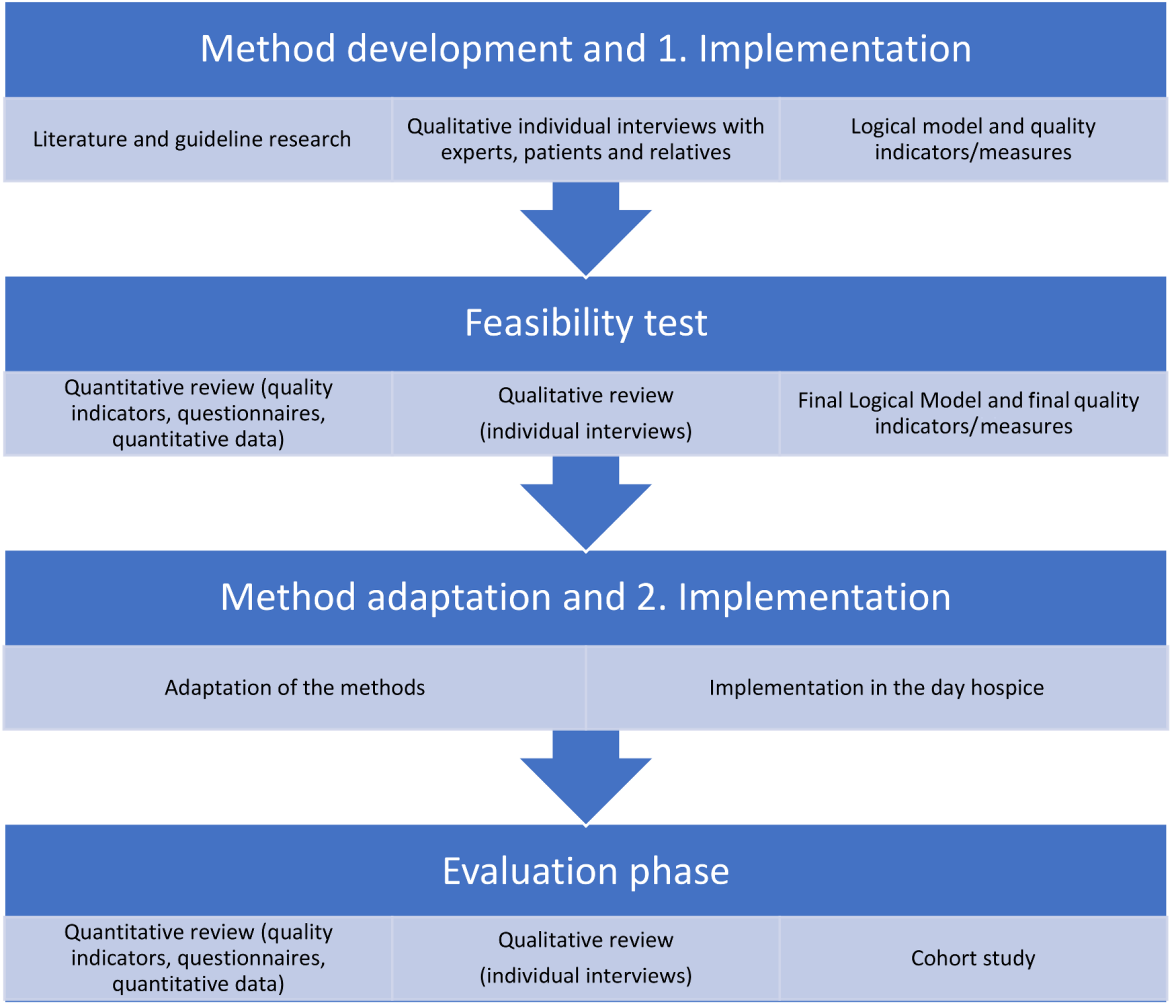
Study design — flowchart.

A prospective, three-stage mixed method study (Fig. 1), guided by the Medical Research Council Framework for the Development and Evaluation of Complex Interventions [16] and the MORECare Statement for the Evaluation of Complex Interventions at the End of Life [17], is planned.

#### Stage 1: Method development (Develop/Identify intervention)

Initially, (a) an extensive literature and guideline research (U1) and (b) qualitative individual interviews [18, 19] (U2) on previous offerings and experiences in day hospices will be conducted. The results are the basis for establishing a first theory model (logical model) [20, 21]. This draft model, including a catalog of organization and offerings, will be cross-checked by participants from U2 and day hospice staff and then finalized. The other aspects of Stage 1 represent the precise definition and development of economic data, metrics/instruments, and quality indicators [22], which will be collected for the reviews of interventions and measures in Stages 2 and 3.

#### Stage 2: Feasibility test (Feasibility)

In Stage 2, the feasibility test of the organization and supply catalog created in Stage 1 and the testing of the metrics and quality indicators under real-life conditions will be performed. Survey variables, structures, and interventions can be continuously adjusted in a dynamic process. Finally, the adjusted measures are cross-checked again by participants from U2 (Stage 1) and day hospice staff, and finalized by the authors.

#### Stage 3: Evaluation phase (Evaluation)

Day hospice organization will be adapted to the final catalog of structures and services developed in Stage 2 and, if necessary, new measures will be implemented. These are then to be tested in an 18-month evaluation phase under real-life everyday conditions in a process test [23] for their feasibility, usefulness, effectiveness, and economic efficiency by using the qualitative and quantitative indicators defined in Stage 2.

### Methods

#### Stage 1: Method development (Develop/Identify intervention)

The usual databases (PubMed, Cochrane Library, Google Scholar, and so on) on day hospice, definition, structure, supply, effect, economics, public relations, and quality indicators, among others, will be used in the planned guideline search in U1.

The qualitative individual interviews in U2 will be conducted with experts (palliative physicians, palliative care nurses, primary care providers from day hospices), guests of the day hospice, and their relatives. In joint consultation with the patient, the relative most directly involved in the care should be invited to participate as the primary caregiver. The interview questions will be based on the study objectives and U1 results. The survey will be conducted in cooperation with “SINUS Markt- und Sozialforschung” by trained and experienced personnel via a secure digital communication medium (either audiovisual or audio only). The planned duration per interview is between 30 and 60 min. The interview place and time are coordinated individually with the participants. The exact number of interviews depends on the goal of generating new data (interviews) until no more new topics are obtained [24]. At least 10 individual interviews will be conducted to achieve sufficient data saturation. The interviews are recorded and transcribed for further processing. For the evaluation, the hermeneutic text interpretation according to Oevermann [18] and the qualitative content analysis according to Mayring [19] are used. Qualitative interviews allow open questions and the possibility for the interviewer to clarify ambiguous responses.

To create a structured and comprehensible theory model including a first structure and offer catalog for the further study steps, we will develop a logical model of a day hospice based on U1 and U2 results [20]. For informed and organized decision making in the research group, the nominal group process is used for this purpose [25].

To develop potential quality indicators, sources from the literature [23, 26, 27] and already defined quality indicators of different guidelines, registers, or indicator databases should serve as a basis [1, 28–30]. The quality indicators are then developed in a structured manner analogous to the S3 guideline for quality indicator development [22] (Table 1).

**Table 1:** Potential parameters for quality indicators

*Patients*	*Relatives*
Quality of life	Quality of life
Social integration and interaction	Relief for family caregivers inpatient care at home
Physical symptom control	Stress reduction for family caregivers
Psychological symptom control	Perceived quality of care of the day hospice
Perceived quality of care of the day hospice	Duration of feasible home care by family caregivers
Duration of a possible antiproliferative therapy (motivation for therapy)	Financial burden
Overall survival	Satisfaction with the care
Number of hospitalizations	
Cost of health care	
Satisfaction with the care	

Quantitative data collection will be based on structured and validated questionnaires or palliative care assessment tools, which are further supplemented by the authors with medical (ECOG, degree of care, and so on), epidemiological, demographic (marital status, residential and social background, and so on), and health economic data (Tables 1 and 2). The health economic evaluation should focus on cost differences in both groups, structural resources of the health care system used (SAPV, nursing services, hospital admissions, medication consumption, and so on), and quality of life [31–33]. For this purpose, a collaboration with the “Allgemeine Ortskrankenkasse Bayern (AOK)” is planned. Before the final release of the collected quantitative data, the questionnaires will still be subjected to a pre-test [34].

**Table 2:** Intended measuring instruments

*Patients*	*Relatives*
EORTC QLQ-C15-PAL [40]	Häusliche Pflege-Skala (HPS-10) [41]
POS – Palliative care Outcome Scale [42] Palliative Care Assessment Questionnaire - Questionnaire for Patients	POS – Palliative care Outcome Scale [42] Palliative Care Assessment Questionnaire - Questionnaire for family caregivers
Disstress Thermometer [43]	Disstress Thermometer [43]
EORTC QLQ-IN-PATSAT32 [44]	EORTC QLQ-IN-PATSAT32 [44]
Minimal documentation system MIDOS to distressing symptoms [45]	QOLLTI-F v2@ - Quality of life during serious illness - family caregivers [46, 47]
Core data set of the national hospice and palliative registry [48]	

#### Stage 2: Feasibility test (Feasibility)

The contents of the (theoretical) offer catalog are established based on the day hospice structures. In a 6-month test phase, the review will then take place:

1. Quantitatively using the measurement tools, target measures, and quality indicators developed in Stage 1. During the test phase, data quality, practicability, and applicability of the planned measurement instruments and quality indicators will be regularly reviewed in daily clinical practice and adjusted if necessary.
2. Qualitative by means of individual participant interviews (patients, relatives, caring medical staff of the day hospice). The qualitative interviews will be conducted regularly during the test phase and analogously to Stage 1. In principle, at least 10 individual interviews are planned, with the questions focusing primarily on the feasibility, acceptance, and implementation possibilities of the structures and services developed in Stage 1 in daily clinical practice.

Based on the data obtained, at the end of the feasibility test, the authors will adapt and finalize the quantitative survey instruments, quality indicators [22], and logical model [20, 21], including the catalog of organizations and services in a renewed nominal group process [25].

#### Stage 3: Evaluation phase (Evaluation)

**Fig. 2:**
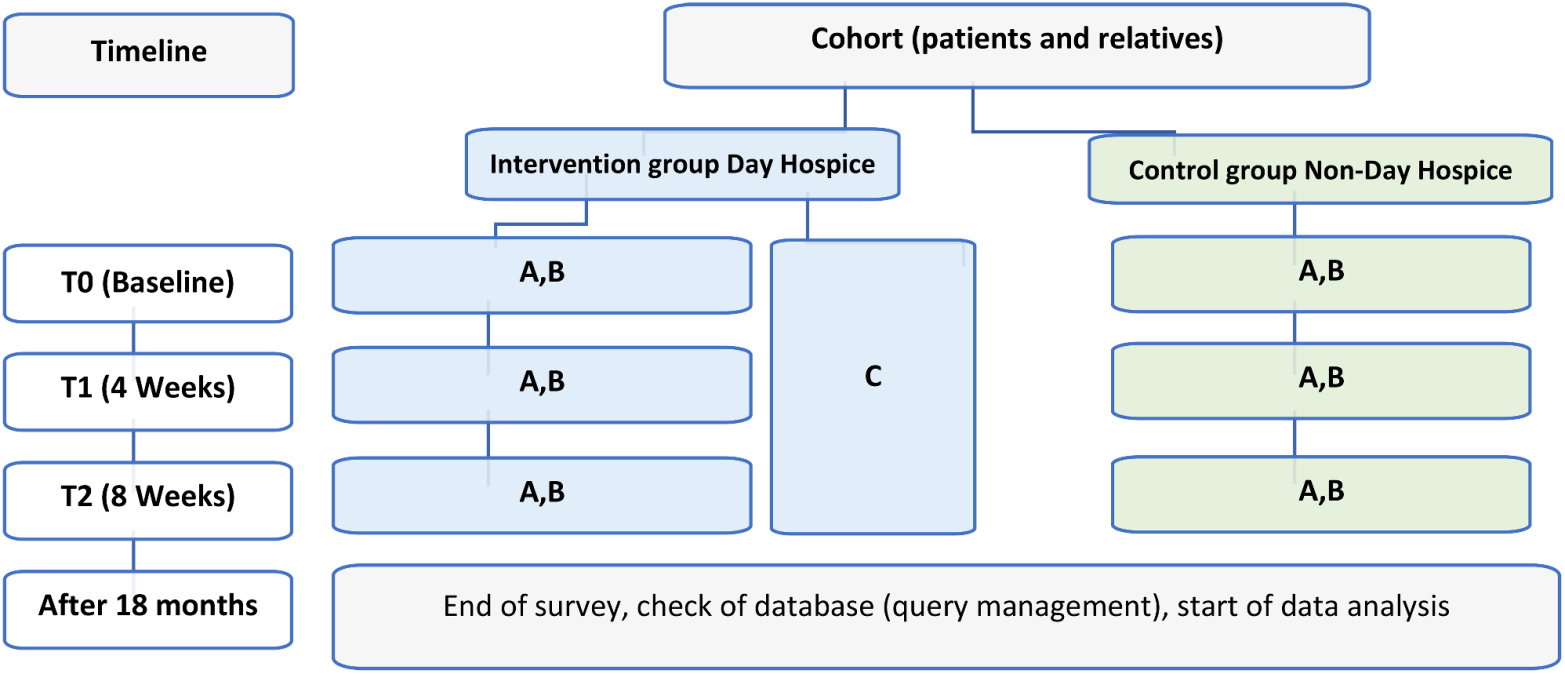
Graphical model of the cohort study [39]. (**A**) Structured questionnaires. (**B**) Structural, epidemiological, demographic, and economic quantitative data. (**C**) Continuous individual qualitative interviews with selected patients and relatives (and staff of the day hospice) throughout the survey phase

The process test will be performed as follows:

1. Use of the quantitative indicators finally defined in Stage 2: structured questionnaires, quality indicators, and medical, epidemiological, demographic, and health economic data.
2. Conduct an initial, baseline, quantitative effectiveness review on the potential benefits of the day hospice. For this purpose, a cohort study [35] with two comparison groups [A — “patients/relatives in the catchment area of the day hospice” (patients living in the catchment area of the day hospice: radius ≤ 25 km approach distance) vs. B — “patients/relatives outside the catchment area of the day hospice” (patients living outside the catchment area of the day hospice: radius > 25 km travel distance)] is planned [12]. The patients (and their relatives) in group A are also cared for in the day hospice, whereas, those in group B are not cared for in the day hospice, but medical care is provided in the same network structure (oncology–palliative care network Landshut, Lower Bavaria, Germany). The oncology–palliative care network Landshut includes several regional hematology–oncology outpatient clinics, an inpatient palliative medicine–oncology care unit, a SAPV, and an inpatient hospice. The network provides for the catchment area of the investigated day hospice and other regions without day hospice facilities to ensure comparability of the two cohorts based on the same oncology and palliative care structures.
  Due to the palliative disease situation of the participants with significantly limited life expectancy, quantitative data will be collected at the measurement time points T0 (baseline, after study inclusion), T1 (after 4 weeks), and T2 (after 8 weeks) (Fig. 2).
3. Individual qualitative interviews will be conducted with the patients and their relatives on an ongoing basis during the evaluation phase in the same way as in Stage 1. Approximately 25–35 interviews are planned, with questions focusing on the perceived benefit from day hospice.

### Recruitment

Identification of appropriate patients and their primary caregiver will be performed in the day hospice by trained staff under the direction of the nursing director, a palliative care specialist (MSc) with an academic degree in applied health services research, or by proxy by study center personnel. The control cohort will be recruited – after appropriate study training – by the physicians of the oncology–palliative care network of Lower Bavaria at the respective locations. The experts for the qualitative interviews are involved by the authors or study leadership team.

### Expected sample size

This study aims to collect real world data and is explorative and observational in character. The sample size is therefore based on feasibility and the case load of the day hospice. The recruitment period for the cohort study will be 18 months. During this period, a consecutive sample of patients meeting the inclusion and exclusion criteria will be included. A caseload of 80 patients with life-limiting illness and an equally large number of the respective relatives in the catchment area of the day hospice (intervention group day hospice) is expected. Likewise, 80 patients with life-limiting illness and an equally large number of the respective relatives outside the catchment area of the day hospice (control group non-day hospice) is expected. Failures are expected in the group of relatives, among other things, due to lack of motivation to participate, patient’s wish, or fundamental lack of relatives. A sample size of 80 patients per group is deemed sufficient according to the literature to estimate the effect of our exploratory endpoints [36, 37].

### Inclusion and exclusion criteria

*Specific admission criteria apply for day hospice visits:*

- Presence of an incurable disease in a very advanced or terminal stage.
- Home care is ensured so that the patient is most likely to return home after visiting the day hospice, and a transfer directly from the day hospice to a full inpatient facility is not necessary.
- The journey to the day hospice is possible in a seated position (e.g., by a transport service or relatives).
- No presence of symptoms that potentially endanger the patients themselves, other guests, or day hospice staff members.
- Ability to fit into the day hospice community and participate in the services/activities offered.
- No complete bedriddenness.
- Age > 18 years.

In principle, admission to the day hospice is always possible regardless of study participation.

*In the present study, the following additional inclusion and exclusion criteria are defined*:

The key inclusion criteria are:

- Care within the oncology–palliative care network Lower Bavaria.
- Presence of severe and life-limiting hematologic/oncologic disease.
- Defined admission criteria for day hospice visit met.

Key exclusion criteria are:

- The absence of care within the oncology–palliative care network Lower Bavaria.
- Inability to answer the questionnaires.
- Defined admission criteria for the visit in the day hospice not met.

*In principle, the inclusion and exclusion criteria can still be modified according to the results of Stages 1 and 2.*

### Data collection, data storage, and data security

To create a protected atmosphere for the participants of the day hospice, the interviews will take place in a separate room in the day hospice. The supervising nurse can always be called, if necessary. A study nurse will also available on site during the interview for technical and organizational questions. Interviews for relatives and experts will take place at the day hospice or participants’ preferred location. The relatives will not be interviewed in the presence of the residents. The data obtained from the interviews will be documented pseudonymously.

All personal data will be handled in accordance with the applicable General Data Protection Regulation (DSGVO).

The medical, epidemiological, and demographic data of the guests of the day hospice are collected via the digital patient file (software system PalliDoc®) and recorded pseudonymously in a central database (eCRF). The questionnaires will be answered in writing by the patients or their relatives, marked pseudonymously, and collected in a sealed box. Within the framework of the cooperation between the oncology–palliative care network Landshut and the University Hospital Regensburg (Clinic and Polyclinic for Internal Medicine III, Center for Palliative Medicine), the analog study documents (including consent forms and questionnaires) will be stored in the archive of the cooperating VK&K Studien GbR. This is a lockable, fire, and waterproof room, to which only trained and documented study center staff have access.

The implementation and programming of the GCP- and DSGVO-compliant database are performed by a data manager from the Center for Clinical Trials Regensburg using the REDCap (Research Electronic Data Capture) system [38] and are hosted at the computer center of the University of Regensburg. REDCap is a browser-based database management system for clinical trials that can perform all essential data management functions (data security, entry, and validation) in a consistent, intuitive, and auditable electronic environment. Validity and consistency checks are programmed into the database. The database will be entered pseudonymously by study nurses on password-protected computers, which are located in lockable rooms of VK&K Studien GbR and are only accessible to their staff members. The Center for Clinical Studies Regensburg is responsible for query management and quality assurance. If inconsistencies exist or corrections to the data are necessary, data queries will be created by the data manager and sent electronically. The data will then be corrected according to the answered and solved queries. After database closure, data are imported into a statistical analysis program and analyzed by a biometrician at the Center for Clinical Trials.

A (pseudonymized) data exchange exists between the institutions conducting the study. For the planned interviews, the study participants’ personal data will be transmitted to the external institute conducting the interviews (SINUS Markt- und Sozialforschung GmbH) or collected by this institute. The participants’ written informed consent must be obtained in advance. Conclusions about the participants can only be drawn by persons directly involved in the study.

### Statistical analysis of quantitative data

The frequency of missing values per variable will be reported. Missing values will not be imputed, unless the handling of missing and ambiguous values will be specified in the manual of the selected measurement instrument. The questionnaires will be evaluated according to the associated manual and questionnaire scores defined in the manual are calculated. A descriptive analysis of the quantitative data will be performed. Depending on the scale level, absolute and relative frequencies will be reported for categorical variables and the mean, standard deviation, median, quartiles, and minimum and maximum for metric variables. Before-and-after comparisons between the survey of the measured variables before (T0, baseline) and after (T1 and T2) the intervention will be performed using appropriate statistical methods. Furthermore, comparisons will be made between the intervention (patients/relatives in the catchment area of the day hospice) and control (patients/relatives outside the catchment area of the day hospice) groups. In the abovementioned analyses, a family of endpoints comprising quality-of-life parameters, quality indicators, and structural and economic parameters is of interest. Due to the exploratory, observational study design, no corrections for multiple testing will be performed. If not defined otherwise, the significance level will be set to p_two−sided_ ≤0.05. Appropriate statistical programs will be used for all analyses, such as SPSS (version 29 or higher) or R (version 4.3.1 or higher). The reasons for departures will be documented.

### Dissemination and implementation

The research data and results of the different research sections will be published in recognized national or international journals and thus made accessible to a broad public. Additionally, the presentations of the research results will be planned at relevant scientific congresses.

## Discussion

To date, initial theoretical recommendations for day hospice implementation in Germany have been published [1, 13]. However, a review on daily clinical practice with systematic and structured implementation development in the sense of a complex intervention [16] and evaluation of the medical-nursing-care and economic effects are still missing. Therefore, for the future establishment of further day hospices in Germany, findings that go beyond theoretical recommendations or analogous conclusions from other countries and health care systems and have been obtained from the real-life situation are essential. Particularly, concrete advice on structural and interventional measures can be greatly beneficial. The planned study is intended to create an initial valid basis for the planning, practical establishment, and concrete implementation of new day hospices based on the real-life clinical care of a day hospice in Germany and/or to identify further necessary optimization processes. Additionally, initial data on the influence of day hospice care on, among other things, the quality of life, coping with illness, and care options of palliative patients and their relatives will be generated.

### Expected results

Specifically, our study results will provide the first real-world data on the feasibility and benefits of structurally developed and tested interventions and structures in a day hospice in Germany, which will include the following

1. Information on structural requirements that is necessary for the establishment and daily operation of a day hospice and that is applicable, feasible, and reasonable in real-life clinical practice for all persons involved in the day hospice (patients, relatives, and caregivers).
2. Development of a final interventional service catalog for day hospices that is tested under clinical conditions and can be implemented in real-life settings.
3. Insights into the economic benefits of day hospices using health economic data.
4. Development of an initial set of potential quality indicators for the (structural and outcome) assessment of day hospices.
5. Insights into the potential benefits of day hospices for patients and relatives in the German healthcare system.

### Study risks

Stages 1 and 2 aimed to develop methods and implement the study investigation. The data collected will be primarily obtained from the literature and interviews with healthcare professionals, patients, their relatives, and experts. The main risk point here will be under-recruitment of interview participants, whether due to lack of interest, lack of time, reduced general health, or skepticism prior to study participation. Due to a good author network with palliative care professionals and experts as well as by a sufficiently large pool of potential patients and relatives from the day hospice, the risk of limited recruitment seems to be justifiably low. In Stage 3, recruitment of patients and their relatives in day hospice is planned for the cohort study. The patients from the oncology–palliative care network Lower Bavaria who are not cared for in the day hospice as well as their relatives are included as a comparison cohort. Particularly, in the comparison cohort, recruitment might therefore prove complicated, e.g., due to a lack of motivation among study participants. Through a total of five different recruitment sites, a sufficiently large number of potential inclusion candidates will be generated to also compensate for larger failures. Additionally, in Stages 1 and 2, an in-depth evaluation of the planned assessment tools and recruitment strategies will be conducted to early identify problematic issues and address them prior to the start of the actual cohort study.

## Conclusions

In our study protocol, we explained the implementation, realization, and benefits of a day hospice in Lower Bavaria of a quality-assured investigation on as well as the structures and interventions to be used. Our study results will be used to facilitate or enable the efficient establishment of day hospices under economic, health, and medical aspects. For the first time, the results of the interventions used and evaluated in real-world day hospice operations will be available. Our data will be used in developing further guidelines for the establishment and operation of day hospices under the aspects of benefit and financing. The present study protocol provides a detailed insight into the intended research objectives, thereby enabling other study groups to perform further investigations. The results of the present study could thus contribute to the further development of palliative care in Germany.

## Data Availability

The datasets that will be used and/or analyzed during the current study are available on reasonable request from the corresponding author.
